# Zoonotic Transmission of Pathogens by *Ixodes ricinus* Ticks, Romania

**DOI:** 10.3201/eid1812.120711

**Published:** 2012-12

**Authors:** Oana Alina Paduraru, Jean Philippe Buffet, Martine Cote, Sarah Bonnet, Sarah Moutailler, Vlad Paduraru, Françoise Femenia, Marc Eloit, Gheorghe Savuta, Muriel Vayssier-Taussat

**Affiliations:** Author affiliations: USC Bipar, Bartonella et Tiques, Maisons-Alfort, France (O.A. Paduraru, J.P. Buffet, M. Cote, S. Bonnet, S. Moutailler, F. Femenia, M. Vayssier-Taussat);; Universitatea de Ştiinţe Agricole şi Medicină Veterinară, Iasi, Romania (O.A. Paduraru, V. Paduraru, G. Savuta);; Pathoquest, Paris, France (O.A. Paduraru, M. Eloit);; Ecole Nationale Vétérinaire d’Alfort, Maisons-Alfort (M. Eloit); and Institut Pasteur, Paris (M. Eloit)

**Keywords:** Ticks, pathogens, human, emerging, Ixodes ricinus, Romania, bacteria, Rickettsiae, Rickettsia, IRS3, monacensis, helvetica, Anaplasma phagocytophilum, Erlichia muris, Babesia, Bartonella, *Neoerhlichia mikurensis*, Borrelia, EU1, Cadiz agent, Rickettsia IRS4, zoonotic transmission, zoonoses, Eyach, viruses

**To the Editor:** The *Ixodes ricinus* tick is a predominant vector of a large variety of pathogens of veterinary and medical consequence in Europe (*1*). The most prevalent *I. ricinus*–borne infection of persons in Europe is Lyme borreliosis, a multisystemic disorder caused by spirochetes of the *Borrelia burgdorferi* sensu lato complex (*2*). Persons bitten by ticks can also become infected with many other pathogens, such as bacteria (*Anaplasma* spp., *Francisella* spp., *Coxiella burnetii*, *Bartonella* spp., *Rickettsiae* spp., and *Neoerhlichia mikurensis*); parasites (*Babesia* spp., *Theileria* spp.); and arboviruses (tick-borne encephalitis virus, Crimean-Congo hemorrhagic fever virus, and Eyach virus) (*1*). Symptoms induced by such pathogens are often diverse and nonspecific, complicating accurate diagnosis of the disease.

In Romania, cases of Lyme disease and tick-borne encephalitis (caused by tick-borne encephalitis virus) have been identified (*3*). However, little is known about the public health impact of these diseases, and none of the other tick-borne pathogens present in Europe have been reported as causes of infection in Romania. Although *I. ricinus* is the most abundant and widespread tick in Romania (*3*), the public health impact of *I. ricinus*–borne disease is likely to be underestimated. Therefore, the first step in evaluating the distribution of these potential pathogens is to establish their presence in ticks from previously unexplored areas.

We conducted a study to identify the main tick-borne bacterial and parasitic human pathogens known to be present in Europe but not previously detected in Romania. We tested for the presence of DNA from spotted fever group *Rickettsia* spp., *Anaplasma* spp., *Francisella tularensis*, and *Babesia* spp. in 147 *I. ricinus* ticks collected from roe deer and goats at 2 sites in eastern Romania: Bacau (46°35′0″N, 26°55′0″E) and Galati (45°26′22″N, 28°2′4″E). Specimens were tested by PCR, using specific primers for each pathogen or group of pathogens, as described (*4*). Sequences obtained from Eurofins MWG Operon (Ebersberg, Germany) were identified by using BLAST (www.ncbi.nlm.nih.gov/BLAST) and compared with sequences available in GenBank. 

DNA from *Rickettsia* spp. was detected in 20 (13.6%) ticks. Sequence analyses revealed that 9 (6.1%) sequences were related to the *R. monacensis* strain IRd/Serbia *glta* gene (99%–100% nt similarity) (GenBank accession no. GQ925820) and 11 (7.48%) were related to *R. helvetica gltA* gene (99%–100% nt similarity) (GenBank accession no. AM418450). DNA from *Anaplasma* spp. was identified in 33 (22.4%) ticks. Analysis revealed that 30 of the 33 amplified fragments showed 100% identity to the 16S rDNA gene of a symbiont in the family *Anaplasmataceae*, *Candidatus *Midichloria mitochondrii (GenBank accession no. EU780455), and the remaining 3 were related to known pathogenic species identified in Romania: 2 (1.4%) exhibited 100% identity to *Anaplasma phagocytophilum* (GenBank accession no. EU982548), and 1 (0.7%) showed 99% similarity to *Ehrlichia muris* (GenBank accession no. GU358691). *Francisella tularensis*–specific DNA was amplified from 4 DNA extracts (2.7%). All 4 sequences were identical and shared 99% similarity with the *F. tularensis* peptidyl-propyl *cis*-*trans* isomerase gene (GenBank accession no. CP003048). *Babesia* spp.–specific DNA was amplified in 1 DNA extract (0.7%), and it shared 99% sequence identity with the *Babesia* sp. EU1 18S rDNA gene (GenBank accession no. HQ830266).

This is a report on the identification of the human pathogens *R. monacensis*, *R. helvetica*, *A. phagocytophilum*, *E. muris,* and *Babesia* EU1 in Romania. *R. monacensis* (also known as the Cadiz agent or *Rickettsia* IRS3 and IRS4) was first identified in *I. ricinus* tick samples from Germany (*5*) and was recently recognized as the cause of Mediterranean spotted fever-like rickettsiosis in Spain (*6*). *R. helvetica* was isolated from *I. ricinus* ticks in Switzerland in 1979, and, since then, it has been isolated in many other European countries (*7*). R. helvetica was associated with human infections in the late 1990s (*8*).

The role of animals as reservoirs for these pathogens is unknown. *A. phagocytophilum* is the causative agent of granulocytic anaplasmosis in humans, cattle, horses, and dogs, and is widespread throughout Europe (*8*). *E. muris* has been detected in ticks from Finland and European Russia and from ticks and rodents from Slovakia. In 2009, E. muris was also detected in patients with febrile illness in the United States (*9*). *Babesia* sp. EU1 has been detected in roe deer and ticks in several countries, and in 2003 was associated with human disease in Italy and Austria (*4*,*10*). Tularemia is known to be present in Romania and is thought to be exclusively and directly transmitted by hares. However, detection of *F. tularensis* DNA in *I. ricinus* ticks suggests that this bacterium might also be tick-borne.

In conclusion, the detection of DNA of various human pathogens in ticks in Romania strongly suggests that these microorganisms circulate in the country. Because all of these pathogens affect humans, our study highlights the urgent requirement for further research to assess their impact on public health in Romania.
